# Effects of weather factors on dengue fever incidence and implications for interventions in Cambodia

**DOI:** 10.1186/s12889-016-2923-2

**Published:** 2016-03-08

**Authors:** Youngjo Choi, Choon Siang Tang, Lachlan McIver, Masahiro Hashizume, Vibol Chan, Rabindra Romauld Abeyasinghe, Steven Iddings, Rekol Huy

**Affiliations:** World Health Organization, Phnom Penh, Cambodia; Australian National University, Canberra, Australia; Nagasaki University, Nagasaki, Japan; National Center for Parasitology, Entomology & Malaria Control, Phnom Penh, Cambodia

**Keywords:** Weather, Dengue, Temperature, Rainfall, Cambodia

## Abstract

**Background:**

Dengue viruses and their mosquito vectors are sensitive to their environment. Temperature, rainfall and humidity have well-defined roles in the transmission cycle. Therefore changes in these conditions may contribute to increasing incidence. The aim of this study was to examine the relationship between weather factors and dengue incidence in three provinces in Cambodia, in order to strengthen the evidence basis of dengue control strategies in this high-burden country.

**Methods:**

We developed negative binomial models using monthly average maximum, minimum, mean temperatures and monthly cumulative rainfall over the period from January 1998 to December 2012. We adopted piecewise linear functions to estimate the incidence rate ratio (IRR) between dengue incidence and weather factors for simplicity in interpreting the coefficients. We estimated the values of parameters below cut-points defined in terms of the results of sensitivity tests over a 0-3 month lagged period.

**Results:**

Mean temperature was significantly associated with dengue incidence in all three provinces, but incidence did not correlate well with maximum temperature in Banteay Meanchey, nor with minimum temperature in Kampong Thom at a lag of three months in the negative binomial model. The monthly cumulative rainfall influence on the dengue incidence was significant in all three provinces, but not consistently over a 0-3 month lagged period. Rainfall significantly affected the dengue incidence at a lag of 0 to 3 months in Siem Reap, but it did not have an impact at a lag of 2 to 3 months in Banteay Meanchey, nor at a lag of 2 months in Kampong Thom.

**Conclusions:**

The association between dengue incidence and weather factors also apparently varies by locality, suggesting that a prospective dengue early warning system would likely be best implemented at a local or regional scale, rather than nation-wide in Cambodia. Such spatial down-scaling would also enable dengue control measures to be better targeted, timed and implemented.

**Electronic supplementary material:**

The online version of this article (doi:10.1186/s12889-016-2923-2) contains supplementary material, which is available to authorized users.

## Background

Dengue fever and severe dengue are mosquito-borne viral diseases of global concern especially in tropical and subtropical regions. According to the World Health Organization (WHO) [1], the disease affects more than 100 countries worldwide, infecting 50-100 million people each year. An estimated 500,000 annual cases of dengue are classified as ‘severe’ and require hospitalization and approximately 2.5 % of these cases are fatal [1]. A large proportion of dengue fever cases occur in children, and many – perhaps a majority – of mild cases are not reported at all. The disease heavily burdens families, communities, health-care systems and inhibits economic growth [2].

Global trends of unplanned urbanization; rising populations; increasing travel and trade; and changes to the physical environment and climate have led to a dramatic resurgence of dengue throughout the regions where suitable vectors are present. In the past five decades the global incidence has increased 30-fold and it will likely continue to escalate [3].

**Fig. 1:**
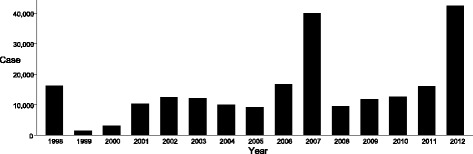
Total number of dengue cases in Cambodia from 1998-2012. Dengue cases: annual total

Cambodia is one of the dengue-endemic countries in South-East Asia, and has been affected by a number of serious epidemics of severe dengue over the last decade. The epidemics appear at intervals of three to seven years, with each epidemic apparently culminating in a higher peak. The most recent epidemic in 2012 saw 42,362 reported cases and 189 deaths (Fig. 1). Transmission occurs mostly during the wet season in Cambodia, with the majority of cases reported between May and November.

It has been suggested that climate change may contribute to an increase in dengue incidence [4–6]. Because dengue viruses and their mosquito vectors are sensitive to their environment, temperature, rainfall and humidity have well-defined roles in the transmission cycle. Therefore changes in these conditions may contribute to increasing incidence [7, 8]. Such conditions include higher temperatures, which can accelerate mosquito development stages and increase dengue transmission, and altered rainfall patterns, producing more standing water – potential breeding sites for mosquitoes [9, 10]. Humidity has been identified as a consistent, substantial weather factor to provide favorable conditions for dengue vectors [11].

Due to the lack of previous studies specific to Cambodia, little is known about the effects of weather factors on the population’s risk of dengue [12]. This study uses statistical analyses to examine the relationship between weather factors and dengue incidence in three provinces in Cambodia, in order to strengthen the evidence basis of dengue control strategies in this high-burden country.

It is intended that the results of this study will help to lay an evidence-based foundation for the implementation of adaptation strategies to reduce the potential for climate change to increase the burden of dengue fever in Cambodia. Ethical approval for this research was granted by the Cambodian Ministry of Health.

## Methods

### Study areas

**Fig. 2:**
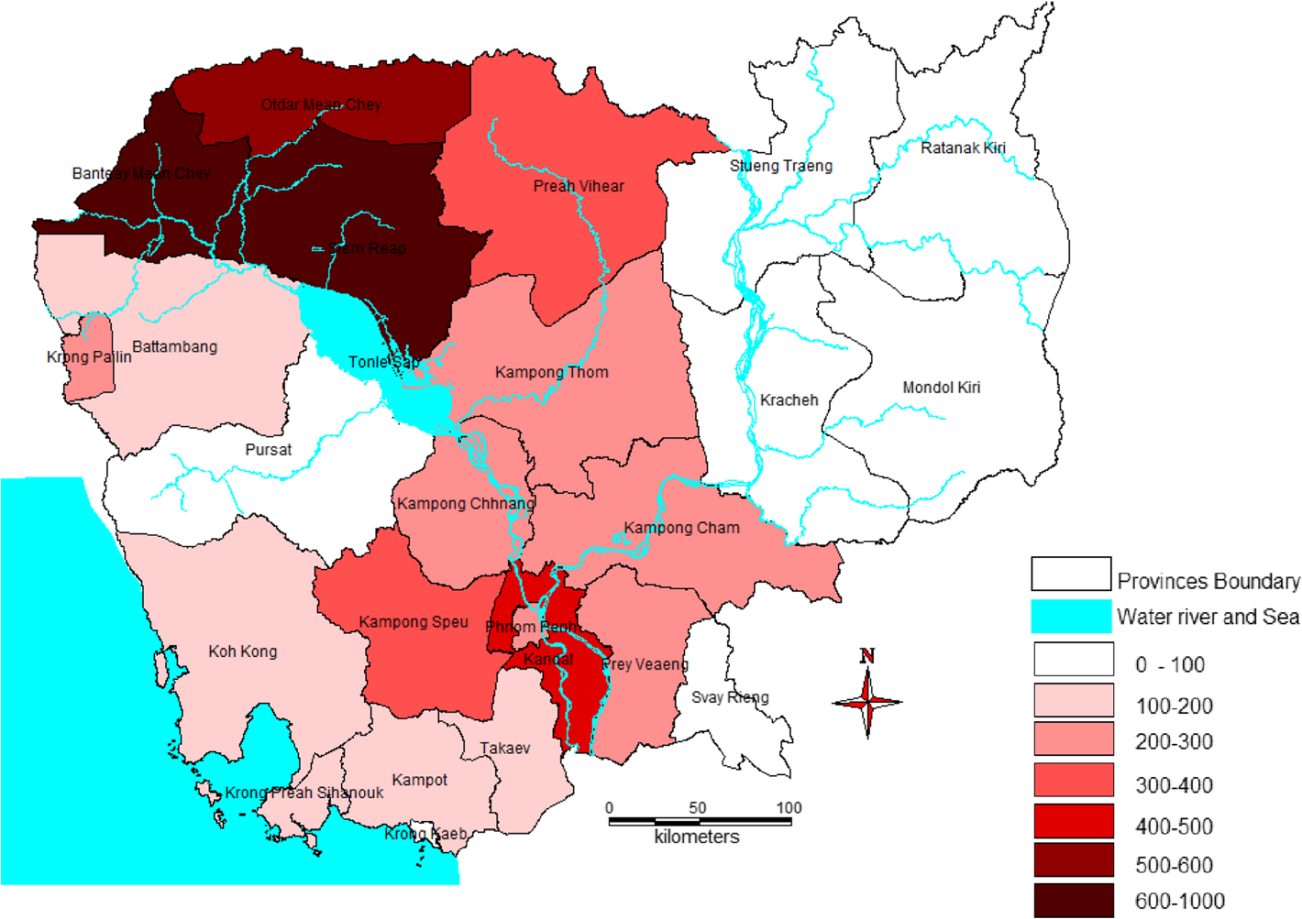
Cumulative incidence of dengue cases per 100,000 population in Cambodia, 2012

The study was conducted in Banteay Meanchey, Kampong Thom and Siem Reap. These three provinces were chosen due to their high burdens of dengue and the availability of relevant dengue and climate data (Fig. 2).

Banteay Meanchey covers an area of 6,679 km^2^; the population was estimated to be 745,328; and the population density was 111 km^2^ in 2012. Kampong Thom covers an area of 13,814 km^2^; the population was estimated to be 694,242; and the population density was 50 km^2^ in 2012. Siem Reap covers an area of 10,299 km^2^; the population was estimated to be 985,650; and the population density was 95 km^2^ in 2012.

### Data

This study utilized fifteen years of data ranging from January 1998 to December 2012 in Banteay Meanchey, Kampong Thom and Siem Reap. The variables included in this study are as follows:

#### Dengue incidence

Monthly number of dengue cases in Banteay Meanchey, Kampong Thom and Siem Reap was supplied by National Dengue Control Program, Ministry of Health, Cambodia;

#### Weather factors

Monthly average minimum, maximum, and mean temperatures as well as monthly cumulative rainfall in Banteay Meanchey, Kampong Thom and Siem Reap weather stations were provided by the Department of Meteorology, Ministry of Water Resources and Meteorology, Cambodia;

#### Population

Population was used as the offset variable in the models. The population data in 1998 and 2008 was collected from National Institute of Statistics, Ministry of Planning, Cambodia, with figures for the remaining years interpolated. The interpolation was performed using the average annual percent change in the population of each province between 1998 and 2008.

### Statistical methods

As a preliminary data analysis, correlation analyses were performed to investigate the relationship between weather factors and dengue incidences. This analysis aimed to identify the optimal lagged effect that various meteorological factors had on dengue incidences (noting that there is typically a lag of weeks to months between changes in weather and associated dengue incidence).

Generalized linear regression models were developed to investigate the association between weather factors and dengue incidence in the three study provinces with and without a time lag. A negative binomial regression model was applied in order to correct for over-dispersion. Mean deviance was used to identify more parsimonious models.

The models incorporated monthly average mean, maximum, and minimum temperature and monthly cumulative rainfall as the independent variables, and the monthly total number of dengue cases as the dependent variable. In order to consider annual population movement, an offset variable of population was used with a logarithmic transformation. Acknowledging that current dengue incidence can be influenced by the number of recent cases (i.e. autocorrelation) [9], a residual autoregressive term of order 1 was included in the models [13], based on autocorrelation function (ACF) and partial autocorrelation function (PACF).

Natural cubic splines of temperature and rainfall with three degrees of freedom per year were included in the models to account for potential non-linear exposure-response relationships between dengue incidence and weather factors. After performing exploratory analyses with natural cubic splines, Fourier terms, and an indicator variable for season, natural cubic spline (3 df) to months (January to December) was incorporated into the models to adjust for seasonal confounding [14]. Indicator variables for the years were also included to account for long term trends and other influences between years, which could confound the associations between weather factors and dengue incidence.

Furthermore, the incidence rate ratio (IRR) between dengue incidence and weather factors was examined to describe the relative risk of the dengue cases in relation to weather factors. A non-linear relationship was observed between dengue incidence and weather factors, hence simple linear regression models were not utilized. Instead, we adopted piecewise linear functions to estimate IRR for simplicity in interpreting the coefficients. We estimated the values of parameters below cut-points defined in terms of the results of sensitivity tests over a 0-3 month lagged period after controlling for seasonal variation, between-year variation, and given weather factors.

The data from the study areas was not combined because the temporal patterns of dengue incidence are quite different between the provinces and the association with different weather variables are also varying between provinces. It is a common and more transparent approach to show the results separately when the association is heterogeneous between the strata/categories [15].

The model details are in the supplementary materials (Additional file 1). Analyses were performed using STATA 13 (Stata Corporation, Texas, USA).

## Results

### Data description

**Fig. 3:**
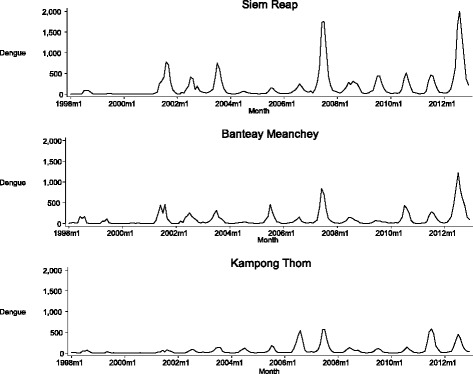
Seasonal variation in dengue cases and weather factors per month from 1998-2012. Dengue cases: monthly total, Rainfall: monthly total (mm) and Temperature: monthly average (°C)

Between 1998-2012, the total number of dengue cases was 17,658, 11,916 and 30,715 in Banteay Meanchey, Kampong Thom and Siem Reap, respectively; and the dengue incidence per 100,000 population was 2,619, 1,868 and 3,500, respectively. Dengue transmission occurred mostly during the wet season between May and October. Figure 3 displays the trend and the descriptive statistics of the variables used for the analyses (Additional file 2: Table S1).

Overall, weather factors showed similar patterns for each season within the same provinces over the years of study. However, there were some differences in temperature and rainfall patterns between the provinces. For instance, the average monthly total rainfall and the average monthly minimum temperature in Banteay Meanchey were higher than in the other two provinces; the average monthly maximum temperature in Siem Reap was higher than the others; and in Kampong Thom, there was poorer correlation between minimum and maximum temperature than elsewhere.

### Correlation analysis

Correlation analyses between the number of dengue cases and climate variables at a lag of zero to three months were performed (Additional file 2: Table S2). The time lag with the highest correlation coefficient was derived from cross- correlation analyses with monthly lags of between zero and seven months. The most significant results were found for lags of 0-3 months, which are presented in this paper.

In all three provinces, the mean temperature, minimum temperature and rainfall were significantly correlated with the number of dengue cases, but maximum temperature was not. Furthermore, the optimal lag time and strengths of correlation with weather factors varied province to province.

### Generalized linear model with negative binomial regression

Negative binomial analyses were performed based on piecewise linear functions after controlling for seasonal variation, between-year variation, and weather factor(s). The Incidence Rate Ratios derive from the generalized linear model (Additional file 2: Tables S3-5).

**Fig. 4:**
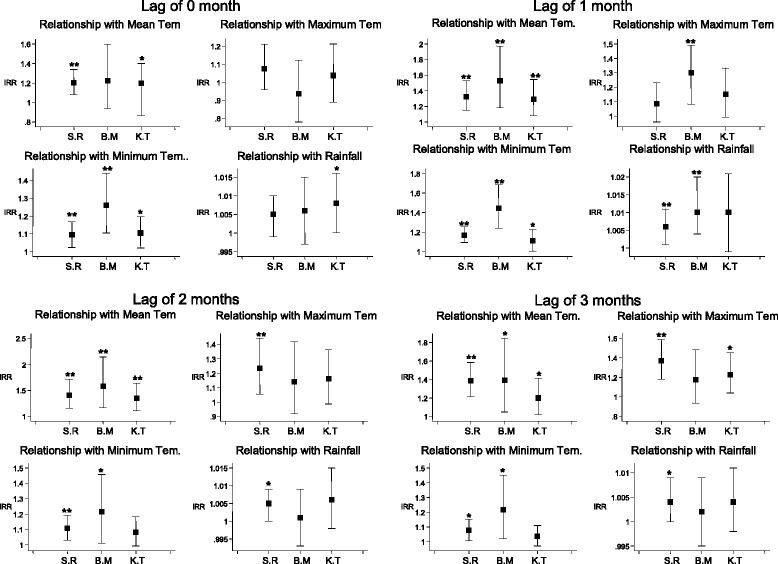
Incidence Rate Ratio of dengue incidence estimated by negative binomial models. S.R: Siem Reap, B.M: Banteay Meanchey, K.T: Kampong Thom. *: *p*-value < 0.05, **: *p*-value < 0.01

Figure 4 describes the change in the number of dengue cases associated with weather factors in all three provinces at a lag of three months. At this lag, a 1 °C increase in average mean temperature led to an increase in the number of dengue cases of 38.6 % (95 % CI: 21.3-58.4), 39.1 % (95 % CI: 4.8-84.5) and 19.9 % (95 % CI: 1.7-41.3) in Siem Reap, Banteay Meanchey and Kampong Thom, respectively. A 1 °C increase in average maximum temperature led to an increase in the number of dengue cases of 36.9 % (95 % CI: 18.1-58.6) and 22.9 % (95 % CI: 4.2-45.1) in Siem Reap and Kampong Thom, respectively. A 1 °C increase in average minimum temperature led to an increase in the number of dengue cases of 7.8 % (95 % CI: 0.8-15.3) and 21.8 % (95 % CI: 2.2-45.1) in Siem Reap and Banteay Meanchey, respectively. For a 1 mm increase in average cumulative rainfall, the number of dengue cases increased by 0.4 % (95 % CI: 0.0-0.9) in Siem Reap; this relationship was not statistically significant in the other provinces.

## Discussion

This paper is the first of its kind in describing the climatic drivers of dengue fever incidence in Cambodia. While many other studies have explored the relationship between factors such as temperature, rainfall and humidity and dengue fever incidence elsewhere in South-East Asia and around the world, the analysis described above provides useful information regarding some key environmental determinants of this potentially fatal vector-borne disease across some of the highest-burden provinces in Cambodia.

The analyses demonstrated a heterogeneous effect of lagged meteorological variables on dengue fever incidence between the provinces. These lag times may be partly accounted for by the inherent delays between weather conditions and their impact on mosquito populations, virus replication and subsequent impact on transmission patterns [16]; however the differences in these lagged effects between provinces requires further exploration.

The monthly average mean, maximum, and minimum temperatures were significantly associated with dengue incidence at a lag of 0 to 3 months in all three provinces. These results were consistent with previous studies in Thailand [9], Puerto Rico [16], Taiwan [17], China [18] and Saudi Arabia [19]. It is known that temperature affects dengue incidence by exerting a sizeable influence on dengue mosquito population dynamics [20]. For example, temperature can have impacts on the conditions for egg-laying, stimulation of egg-hatching and the abundance of *Aedes* larvae and pupae [21–23]. These ecological processes lead to larger *Aedes* mosquito populations, more rapid viral replication, greater transmission risk, and increased dengue incidence. The precise effects of temperature on dengue incidence are somewhat inconsistent in the literature [19, 24–26].

Mean temperature was significantly associated with dengue incidence in all three provinces, but incidence did not correlate well with maximum temperature in Banteay Meanchey, nor with minimum temperature in Kampong Thom at a lag of three months in the negative binomial model.

The monthly cumulative rainfall influence on the dengue incidence was significant in all three provinces, but not consistently, over a 0-3 month lagged period. Rainfall significantly affected the dengue incidence at a lag of 0 to 3 months in Siem Reap, but it did not have an impact at a lag of 2 to 3 months in Banteay Meanchey, nor at a lag of 2 months in Kampong Thom. Similarly heterogeneous effects of rainfall on dengue incidence have been shown in Brazil [24], Vietnam [25], Philippines [27] and Trinidad [28].

The effect of rainfall on dengue incidence is exerted via complex pathways, interacting with other meteorological factors and environmental parameters. Higher rainfall has been associated with dengue in Puerto Rico [16], Taiwan [17], Barbados [29], Indonesia [30], Mexico [31], Thailand [32], Trinidad [33], and Venezuela [34]. Rainfall events, generally, can create breeding habitats for juvenile *Aedes* mosquitoes and subsequently lead to increasing mosquito abundance. However, mosquito abundance and breeding habitats can be destroyed by a heavy spell of rainfall, as it can flush away larvae and pupae from breeding sites [35–37]. Conversely, decreased rainfall can increase mosquito abundance as households increase the use of water storage receptacles [38, 39].

Spatial heterogeneity in the association between rainfall and the incidence of dengue has been attributed elsewhere to local topographic conditions and vector populations and species [16]. In order to investigate the effect of rainfall, interacting with other weather conditions, further studies incorporating *Aedes* population measurements with other weather factors such as humidity, water evaporation, wind speed and cloud cover are required. In addition, the effects of changing rainfall on society and human behaviors need to be considered as such social and behavioral factors can influence *Aedes* mosquito population dynamics, for example by leading to differences in water storage practices or activity patterns [9].

The limitations of this study include the lack of incorporation of demographic, ecological and socio-economic factors in the analysis, which may be expected to significantly improve the robustness of the models. This is subject of ongoing research in the scale-up and implementation of climate change and health adaptation work in Cambodia

In Cambodia, as dengue spreads from urban settings to more rural areas, cases of severe dengue are being reported from increasingly remote locations. It is thought that human movement, particularly via road traffic, is a major factor in the propagation of dengue epidemics in Cambodia [40]. Consequently, the population at risk has increased from the 3.5 million people living in the urban areas to almost 11 million people in a wider geographic range [41]. The magnitude of the public health problem in the country will continue to grow unless more effective measures are taken to reduce dengue transmission.

Currently, there is still no vaccine or specific treatment available against the virus. Hence, vector control remains one of principle strategies for reducing dengue transmission. Effective integrated vector management (IVM) relies on vector surveillance and control, environmental management, community participation, and health education. However, IVM is resource intensive and expensive to implement, especially in a developing country like Cambodia.

## Conclusions

This study suggests that meteorological factors such as temperature and rainfall are significantly associated with dengue fever incidence in some regions of Cambodia, at time lags of up to three months. This window of opportunity, in advance of the peak dengue transmission periods, may provide sufficient time to mobilize resources to implement intervention measures to minimize the epidemic impact.

The association between dengue incidence and weather factors also apparently varies by locality, suggesting that a prospective dengue early warning system would likely be best implemented at a local or regional scale, rather than nation-wide. Such spatial down-scaling would also enable dengue control measures to be better targeted, timed and implemented. The knowledge gained from the current study is also potentially applicable to neighboring countries, which share many of Cambodia’s weather, environmental conditions and social conditions.

Further study on other weather parameters should also conducted, so that a more sophisticated early warning system model for dengue epidemic prediction can be developed, as part of a broader suite of measures aimed at protecting the health of Cambodian communities in the face of emerging infectious diseases and climate change.

## Additional files

Additional file 1: Model details. (DOC 30 kb) Additional file 2: Table S1-S5.. (DOC 97 kb)
