# Supplementary material for: Effects of weather factors on dengue fever incidence and implications for interventions in Cambodia
Source: BMC Public Health. 2016 Mar 8;16:241. doi: 10.1186/s12889-016-2923-2 (PMC4784273; doi:10.1186/s12889-016-2923-2)
Supplement: Additional file 1: — Model details. (DOC 30 kb) [file 12889_2016_2923_MOESM1_ESM.doc]

**· Model details**

The following function was used for generalized linear models:

Log*e* (*dengue cases*) = β0 + β1*s*(*temperature, 3df/year*)t-*i* + β2*s*(*rainfall, 3df/year*)t-*i* + *i*.*year* + *s*(*month, 3df/year*) + *offset(population)* + *ar*

In the above function, β_0_ is the constant, β_1_-β_2_ are the unknown parameter values to be estimated and *i* is a given lag-time; *s* represents a natural cubic spline function; *i.year* denotes indicator variables for the years; *month* represents a calendar month; *ar* indicates autoregressive terms. The model does not include mean, minimum and maximum temperatures simultaneously.
