# Supplementary material for: Effects of weather factors on dengue fever incidence and implications for interventions in Cambodia
Source: BMC Public Health. 2016 Mar 8;16:241. doi: 10.1186/s12889-016-2923-2 (PMC4784273; doi:10.1186/s12889-016-2923-2)
Supplement: Additional file 2: — Table S1-S5.. (DOC 97 kb) [file 12889_2016_2923_MOESM2_ESM.doc]

Table S1. Basic statistics of the monthly local climate variables and dengue cases per month from 1998 to 2012

| Variable (unit) |  | Siem Reap | Banteay Meanchey | Kampong Thom |
| --- | --- | --- | --- | --- |
| Mean  Temperature (°C) | Min | 23.80 | 23.30 | 23.50 |
|  | Max | 33.00 | 32.40 | 32.30 |
|  | Mean | 28.43 | 28.46 | 27.54 |
| Maximum  Temperature (°C) | Min | 31.2 | 29.40 | 28.80 |
|  | Max | 41.5 | 40.30 | 40.20 |
|  | Mean | 35.25 | 33.50 | 33.37 |
| Minimum  Temperature (°C) | Min | 10.7 | 15.00 | 13.00 |
|  | Max | 26.5 | 26.90 | 28.00 |
|  | Mean | 20.97 | 23.38 | 21.66 |
| Rainfall (mm) | Min | 0 | 0 | 0 |
|  | Max | 513 | 452.20 | 497.20 |
|  | Mean | 121.20 | 103.97 | 129.47 |
| Dengue Cases | Min | 0 | 0 | 0 |
|  | Max | 2006 | 1224 | 584 |
|  | Mean | 170 | 98 | 66 |
| Dengue Cases  per 100,000 pop. | Min | 0 | 0 | 0 |
|  | Max | 204 | 164 | 92 |
|  | Mean | 19 | 14 | 10 |

Table S2. Correlation analysis between the number of dengue cases and climate variables with time lag effects in three Provinces

| Climate Variable | Lag Time | Siem Reap | Banteay Meanchey | Kampong Thom |
| --- | --- | --- | --- | --- |
| Mean Temperature | 0 | 0.200** | 0.216** | 0.246** |
|  | 1 | 0.398** | 0.417** | 0.380** |
|  | 2 | 0.4915** | 0.443** | 0.413** |
|  | 3 | 0.455** | 0.370** | 0.301** |
| Maximum Temperature | 0 | -0.09 | 0.017 | 0.129 |
|  | 1 | 0.166* | 0.289** | 0.307** |
|  | 2 | 0.363** | 0.469** | 0.438** |
|  | 3 | 0.455** | 0.545** | 0.495** |
| Minimum Temperature | 0 | 0.399** | 0.295** | 0.219** |
|  | 1 | 0.486** | 0.357** | 0.207** |
|  | 2 | 0.429** | 0.237** | 0.108 |
|  | 3 | 0.272** | 0.060 | -0.070 |
| Rainfall | 0 | 0.366** | 0.277** | 0.361** |
|  | 1 | 0.304** | 0.195** | 0.256** |
|  | 2 | 0.270** | 0.154* | 0.201** |
|  | 3 | 0.011 | -0.154* | -0.083 |

Spearman rank correlation and Pearson correlation analyses were performed with temperature and rainfall respectively.

*: p-value<0.05, **: p-value<0.01,

Table S3. Incidence Rate Ratio (IRR) in Siem Reap

| Lag | 0 month | | 1 month | | 2 months | | 3 months | |
| --- | --- | --- | --- | --- | --- | --- | --- | --- |
|  | IRR | CI | IRR | CI | IRR | CI | IRR | CI |
| Mean Tem. | 1.202** | 1.080-1.338 | 1.327** | 1.154-1.526 | 1.406** | 1.159-1.706 | 1.386** | 1.213-1.584 |
| Max Tem. | 1.076 | 0.958-1.209 | 1.085 | 0.959-1.230 | 1.234** | 1.055-1.443 | 1.369** | 1.181-1.586 |
| Min Tem. | 1.094** | 1.023-1.169 | 1.159** | 1.094-1.255 | 1.108** | 1.030-1.192 | 1.078* | 1.008-1.153 |
| Rainfall | 1.005 | 0.999-1.010 | 1.006** | 1.001-1.011 | 1.005* | 1.000-1.009 | 1.004* | 1.000-1.009 |

CI represents confidence interval

Cut-point: mean temperature 29.80°C; maximum temperature 37.5°C; minimum temperature 24°C; rainfall 135.40mm

*: p-value < 0.05, **: p-value < 0.01

Table S4. Incidence Rate Ratio (IRR) in Banteay Meanchey

| Lag | 0 month | | 1 month | | 2 months | | 3 months | |
| --- | --- | --- | --- | --- | --- | --- | --- | --- |
|  | IRR | CI | IRR | CI | IRR | CI | IRR | CI |
| Mean Tem. | 1.223 | 0.936-1.595 | 1.527** | 1.183-1.972 | 1.583** | 1.170-2.140 | 1.391* | 1.048-1.845 |
| Max Tem. | 0.935 | 0.780-1.121 | 1.301** | 1.083-1.492 | 1.142 | 0.919-1.420 | 1.178 | 0.937-1.481 |
| Min Tem. | 1.260** | 1.104-1.439 | 1.445** | 1.239-1.686 | 1.213* | 1.010-1.457 | 1.218* | 1.022-1.451 |
| Rainfall | 1.006 | 0.997-1.016 | 1.011** | 1.004-1.020 | 1.001 | 0.993-1.009 | 1.002 | 0.995-1.009 |

CI represents confidence interval

Cut-point: mean temperature 28°C; maximum temperature 35°C; minimum temperature 23°C; rainfall 97mm

*: p-value < 0.05, **: p-value < 0.01

Table S5. Incidence Rate Ratio (IRR) in Kampong Thom

| Lag | 0 month | | 1 month | | 2 months | | 3 months | |
| --- | --- | --- | --- | --- | --- | --- | --- | --- |
|  | IRR | CI | IRR | CI | IRR | CI | IRR | CI |
| Mean Tem. | 1.200* | 0.865-1.399 | 1.292** | 1.082-1.544 | 1.351** | 1.115-1.637 | 1.199* | 1.017-1.413 |
| Max Tem. | 1.037 | 0.888-1.212 | 1.150 | 0.992-1.333 | 1.160 | 0.987-1.363 | 1.229* | 1.042-1.451 |
| Min Tem. | 1.105* | 1.020-1.196 | 1.112* | 1.004-1.230 | 1.083 | 0.992-1.183 | 1.038 | 0.971-1.110 |
| Rainfall | 1.008* | 1.000-1.016 | 1.010 | 0.999-1.021 | 1.006 | 0.998-1.015 | 1.004 | 0.998-1.011 |

CI represents confidence interval

Cut-point: mean temperature 29°C; maximum temperature 35°C; minimum temperature 24°C; rainfall 95mm

*: p-value < 0.05, **: p-value < 0.01
